# Supplementary material for: Effect of Different Edible Trichosanthes Germplasm on Its Seed Oil to Enhance Antioxidant and Anti-Aging Activity in Caenorhabditis elegans
Source: Foods. 2024 Feb 5;13(3):503. doi: 10.3390/foods13030503 (PMC10855050; doi:10.3390/foods13030503)
Supplement: Supplementary file 1 [file foods-13-00503-s001.zip › Supplementary Table S1.pdf]

Table S1. Effects of the seed oils of from 18 edible *Trichosanthes* germplasm on oxidative stress tolerance in *C. elegans*

| Groups | Mean lifespan (h) (Mean ± SD) | Maximum lifespan (h) (Mean ± SD) | Mean fold Change (%) |
|---|---|---|---|
| YNHH | 22.18±0.73 $^{A}$ | 52.00±0.00 | 37.76 |
| HNXX | 21.96±0.22 $^{AB}$ | 52.00±0.00 | 36.40 |
| HNZZ | 21.68±0.74 $^{AB}$ | 52.00±0.00 | 34.66 |
| GXYL | 21.59±0.58 $^{ABC}$ | 48.00±0.00 | 34.10 |
| JXJJ | 21.55±0.36 $^{ABC}$ | 52.00±0.00 | 33.85 |
| AHAQ | 21.47±0.75 $^{A-D}$ | 48.00±0.00 | 33.35 |
| JSXZ | 21.20±0.38 $^{A-D}$ | 48.00±0.00 | 31.68 |
| Linseed oil | 20.88±0.33 $^{B-E}$ | 49.33±1.89 | 29.69 |
| HNLY | 20.87±0.58 $^{B-E}$ | 48.00±0.00 | 29.63 |
| SCNC | 20.80±0.10 $^{B-E}$ | 48.00±0.00 | 29.19 |
| HBBD | 20.42±0.19 $^{CDE}$ | 44.00±0.00 | 26.83 |
| SDJN | 20.28±0.26 $^{DE}$ | 48.00±0.00 | 25.96 |
| ZJPH | 19.83±0.30 $^{EF}$ | 44.00±0.00 | 23.17 |
| SXHZ | 19.79±0.37 $^{FG}$ | 44.00±0.00 | 22.92 |
| GZGY | 18.81±0.18 $^{FG}$ | 44.00±0.00 | 16.83 |
| HBSY | 18.48±0.32 $^{G}$ | 40.00±0.00 | 14.78 |
| ZJCX | 18.41±0.78 $^{G}$ | 40.00±0.00 | 14.35 |
| ZJQT | 18.31±0.38 $^{G}$ | 40.00±0.00 | 13.73 |
| FJNP | 18.09±0.63 $^{G}$ | 40.00±0.00 | 12.36 |
| Blank Control | 16.10±0.41 $^{H}$ | 36.00±0.00 | / |
| DMSO | 15.94±0.37 $^{H}$ | 36.00±0.00 | -0.99 |

The data were analyzed by one way-ANOVA analysis and different uppercases indicated significant difference at level of 0.01 by Least-Significant Difference Test (LSD).

Red: *T. laceribractea* Hayata; Blue: *T. rosthornii* Harms; Green: *T. kirilowii* Maxim.
