# Supplementary material for: Effect of Different Edible Trichosanthes Germplasm on Its Seed Oil to Enhance Antioxidant and Anti-Aging Activity in Caenorhabditis elegans
Source: Foods. 2024 Feb 5;13(3):503. doi: 10.3390/foods13030503 (PMC10855050; doi:10.3390/foods13030503)
Supplement: Supplementary file 1 [file foods-13-00503-s001.zip › Supplementary Table S2.pdf]

Table S2. Effects of the seed oils from selected edible *Trichosanthes* germplasm on the longevity of *C. elegans*.

Groups	Mean lifespan (h) (Mean ± SD)	Maximum lifespan (h) (Mean ± SD)	Mean fold Change (%)
SDJN	22.17±0.61 A	34.00±0.00	45.46
YNHH	22.15±0.65 A	34.00±0.00	45.31
GXYL	20.80±0.74 A	32.00±0.00	36.44
SXHZ	18.98±0.42 B	28.00±0.00	24.54
Linseed oil	18.09±0.72 B	26.00±0.00	18.65
ZJQT	17.59±0.40 B	24.00±0.00	15.40
Blank Control	15.24±0.65 C	24.00±0.00	/

The data were analyzed by one way-ANOVA analysis and different uppercases indicated significant difference at level of 0.01 by Least-Significant Difference Test (LSD).

Red: *T. laceribractea* Hayata; Blue: *T. rosthornii* Harms; Green: *T. kirilowii* Maxim.
