# Supplementary material for: Effect of Different Edible Trichosanthes Germplasm on Its Seed Oil to Enhance Antioxidant and Anti-Aging Activity in Caenorhabditis elegans
Source: Foods. 2024 Feb 5;13(3):503. doi: 10.3390/foods13030503 (PMC10855050; doi:10.3390/foods13030503)
Supplement: Supplementary file 1 [file foods-13-00503-s001.zip › Supplementary Table S3.pdf]

Table S3. Effects of the seed oils from selected edible *Trichosanthes* germplasm on the lipofuscin accumulation of *C. elegans*.

**Groups**	**Relative lipofuscin fluorescence**	**Change (%)**
YNHH	0.30 ± 0.02 ^{F}	- 70.35
Positive control	0.45 ± 0.01 ^{E}	- 54.65
SDJN	0.53 ± 0.01 ^{D}	- 47.11
GXYL	0.63 ± 0.01 ^{C}	- 37.30
ZJQT	0.75 ± 0.00 ^{B}	- 25.31
SXHZ	0.94 ± 0.01 ^{A}	- 2.27
Blank Control	1.00 ± 0.00 ^{A}	/

The data were analyzed by one way-ANOVA analysis and different uppercases indicated significant difference at level of 0.01 by Least-Significant Difference Test (LSD).

Red: *T. laceribractea* Hayata; Blue: *T. rosthornii* Harms; Green: *T. kirilowii* Maxim.
