# Supplementary material for: Effect of Different Edible Trichosanthes Germplasm on Its Seed Oil to Enhance Antioxidant and Anti-Aging Activity in Caenorhabditis elegans
Source: Foods. 2024 Feb 5;13(3):503. doi: 10.3390/foods13030503 (PMC10855050; doi:10.3390/foods13030503)
Supplement: Supplementary file 1 [file foods-13-00503-s001.zip › Supplementary Table S4.pdf]

Table S4. Effects of the seed oils from selected edible *Trichosanthes* germplasm on the body bending and pharyngeal pumping rates of *C. elegans* at different stages.

Groups	Adult age	Number of body bending in 30s	Number of pharyngeal pumping in 15s
YNHH	Day 3	18.87±0.63 A	59.73±0.76 A
SDJN		18.47±0.34 A	55.43±0.21 B
GXYL		18.18±0.09 AB	53.60±0.41 C
Linseed oil		17.63±0.42 B	51.40±0.59 D
SXHZ		17.53±0.21 B	49.50±0.08 E
ZJQT		12.53±0.12 C	47.97±0.37 F
Blank Control		12.43±0.12 C	47.83±0.61 F
YNHH	Day 7	13.83±0.09 A	46.13±0.12 A
SDJN		13.20±0.62 AB	45.07±0.12 B
GXYL		12.43±0.34 BC	44.57±0.34 BC
SXHZ		12.17±0.17 C	43.77±0.25 D
Linseed oil		12.13±0.25 C	44.20±0.22 CD
ZJQT		8.67±0.21 D	41.03±0.33 E
Blank Control		8.27±0.34 D	40.73±0.21 E

The data were analyzed by one way-ANOVA analysis and different uppercases indicated significant difference at level of 0.01 by Least-Significant Difference Test (LSD).

Red: *T. laceribractea* Hayata; Blue: *T. rosthornii* Harms; Green: *T. kirilowii* Maxim.
