# Supplementary figures and images for: Effect of Different Edible Trichosanthes Germplasm on Its Seed Oil to Enhance Antioxidant and Anti-Aging Activity in Caenorhabditis elegans
Source: Foods. 2024 Feb 5;13(3):503. doi: 10.3390/foods13030503 (PMC10855050; doi:10.3390/foods13030503)

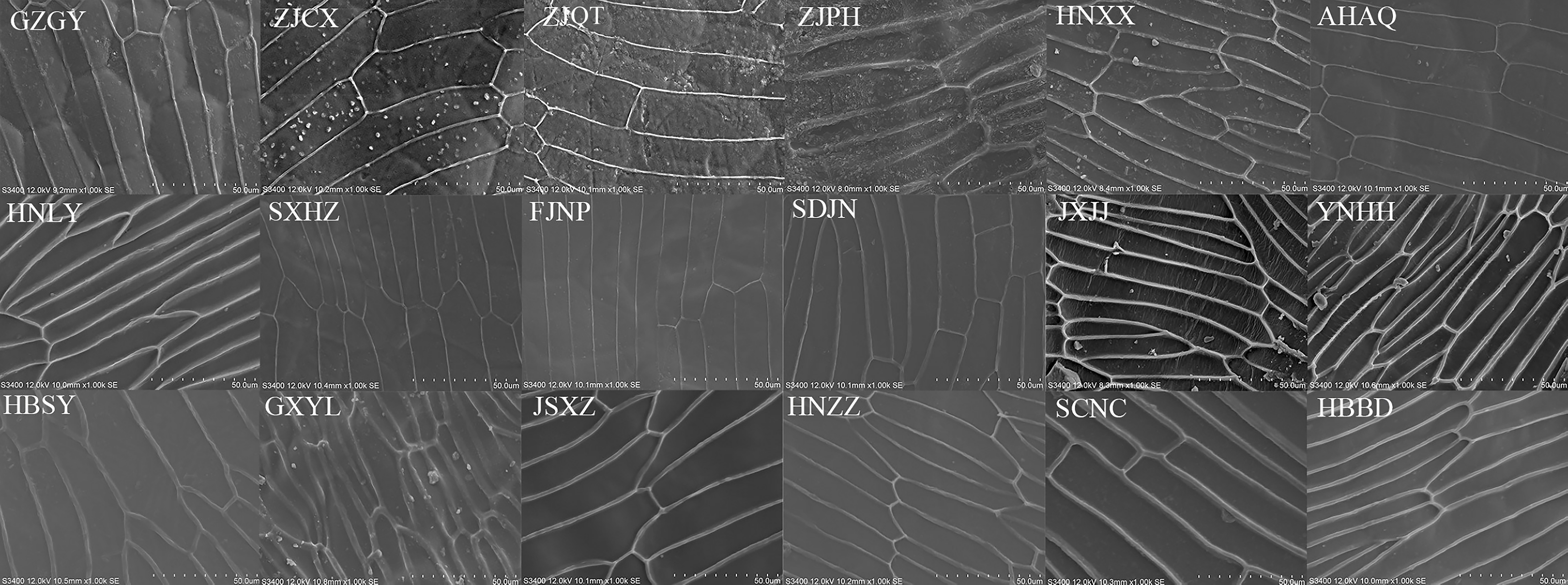

Supplement: Supplementary file 1 [file foods-13-00503-s001.zip › Supplementary Figure S1.jpg]
